# Molecular dynamics study on the mechanism of polynucleotide encapsulation by chitosan

**DOI:** 10.1038/s41598-017-05197-0

**Published:** 2017-07-11

**Authors:** Jia-Wei Shen, Jiachen Li, Zhennan Zhao, Li Zhang, Guoteng Peng, Lijun Liang

**Affiliations:** 1 School of Medicine, Hangzhou Normal University, Hangzhou, 310016 People’s Republic of China; 2 Department of Chemistry, Key Laboratory of Advanced Textile Materials and Manufacturing Technology of Education Ministry, Zhejiang Sci-Tech University, Hangzhou, 310018 People’s Republic of China; 3 College of Life Information Science and Instrument Engineering, Hangzhou Dianzi University, Hangzhou, 310018 People’s Republic of China

## Abstract

The safe and effective delivery of therapeutic genes into target cell interiors is of great importance in gene therapy. Chitosan has been extensively studied as a gene delivery carrier due to its good biocompatibility and biodegradability. Understanding the atomic interaction mechanism between chitosan and DNA is important in the design and application of chitosan-based drug and gene delivery systems. In this work, the interactions between single-stranded polynucleotides and different types of chitosan were systematically investigated by using molecular dynamics (MD) simulation. Our results demonstrate that the functional groups of chitosan, the types of base and length of polynucleotides regulate the interaction behavior between chitosan and polynucleotides. The encapsulation capacity of polynucleotide by chitosan is mainly balanced by two factors: the strength of polynucleotide binding to chitosan and the tendency of self-aggregation of polynucleotide in the solution. For –NH_3_^+^ chitosan, due to the strong electrostatic interaction, especially the H-bond between –NH_3_^+^ groups in chitosan and phosphate groups in polynucleotide, the aggregation effect could be partially eliminated. The good dispersal capacity of polynucleotides may improve the encapsulation of polynucleotides by chitosan, and hence increase the delivery and transfection efficiency of chitosan-based gene carrier.

## Introduction

The safe and effective delivery of therapeutic genes into target cell interiors is of great importance in gene therapy^1–5^. Although the traditional viral vectors can offer better efficiency of gene delivery, due to its side effect (e.g., endogenous virus recombination, oncogenic effects, unexpected immune response etc.) on clinical applications^6–8^, it has been gradually replaced by non-viral vectors (i.e., typically based on polymeric micelles, dendrimers, nanoparticles, liposomes etc.)^9–11^.

**Figure 1:**
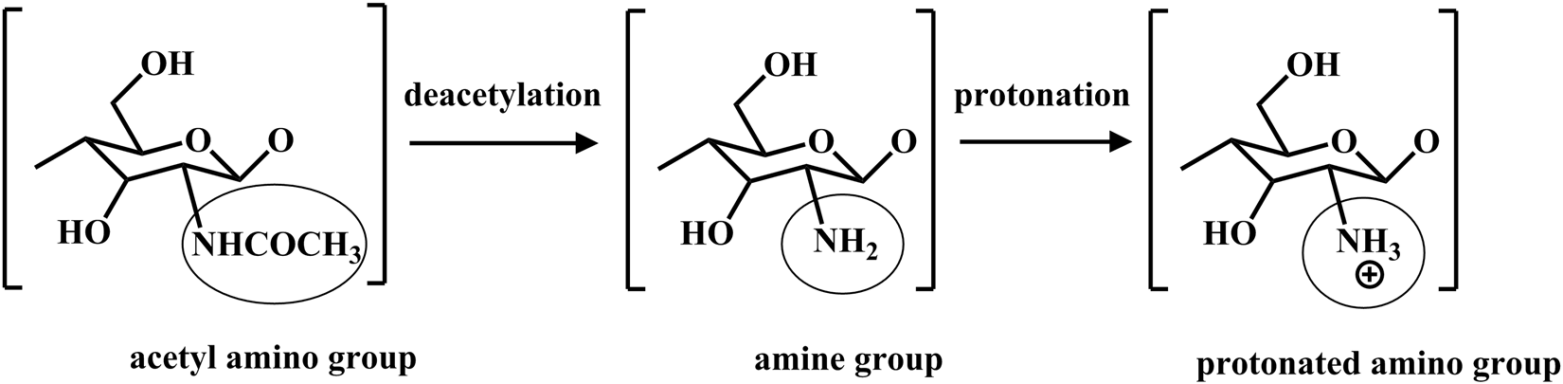
The schemetic of chitosan’s versatility with different functional groups.

As a member of non-viral vectors, much attention have been given to chitosan and chitosan derivatives due to their biodegradability, biocompatibility and their mucoadhesive as well as high positive charge density in low pH solution^6, 12–14^. As shown in Fig. 1, chitosan can be obtained by deacetylation of chitin, and the primary amines in the chitosan backbone can subsequently become positively charged in low pH solution. Its positively charged character enables chitosan binding strongly to several mammalian cells^15^. Moreover, chitosan forms polyelectrolyte complexes with negatively charged DNA, in which the DNA becomes better protected against nuclease degradation^16^. These beneficial characters give chitosan the abilities to be a competent gene carrier. However, the gene delivery and transfection efficiency of chitosan is relatively low, thus their clinical applications were greatly limited^2^.

Further studies also showed that the transfection efficiency of chitosan is significantly influenced by a series of chitosan-based formulation parameters, such as the molecular weight (MW) of chitosan, its degree of deacetylation (DD), the charge ratio of chitosan to DNA/siRNA etc.^17–21^. For instance, the study of Lavertu *et al*.^21^ has shown that the maximum transfection efficiency could be obtained by simultaneously increasing the MW and decreasing the DD, indicating a multi-factor effect (i.e., particle stability and electrostatic binding of chitosan-DNA complexes) in determining transfection efficiency. There are also some studies reported the modification of chitosan for the purpose of improving transfection efficiency^22, 23^. Gao *et al*.^22^ prepared an arginine-chitosan gene carrier where the arginine was linked through NH_2_ group. They concluded that modification of functional groups on chitosan could improve chitosan’s water solubility and enhance its gene transfection efficiency in HEK 293 and COS-7 cells. Guţoaia *et al*.^23^ recommended that fine-tuned PEGylation of chitosan can be used to generate PEG-chitosan/siRNA delivery systems with maximum bioactivity. These experiments greatly enhanced our understanding of the property of chitosan as a gene carrier. In addition, all of these experiments, including chitosan-based formulation parameters and chitosan modification show that the purposeful changing of functional group in the backbone of chitosan may provide an effective strategy in improving the transfection efficiency. However, the interaction mechanism between chitosan and DNA is still unclear at molecular level. In particular, the effect of functional group in the backbone of chitosan (e.g., –NH_2_, –NH_3_^+^ and –NHCOCH_3_, as shown in Fig. 1) on DNA delivery is still obscure.

In fact, with the goal of improving the transfection efficiency and realizing real application of chitosan-based gene systems, it is of great importance to investigate the effect of functional group on DNA delivery and underlying mechanism in details. Recently, with the help of fast-growing computational power, molecular dynamics (MD) simulation may offer plenty of useful information on this challenge by shedding light onto the atomic level details of chitosan-based drug/gene delivery systems^24, 25^. Recently, MD simulation have been widely used to study the critical steps in gene delivery, including nanoparticle formation dynamics with various carriers, complexation of carriers with polynucleotides, carrier conformations in endosome stages, and release of polynucleotides from carriers etc.^26–32^. For example, Zhan *et al*.^31^ reported that the hydrophobic/hydrophilic modifications of linear polycations may bring improvement on its properties as gene carrier. Ding *et al*.^32^ found that the property of polyelectrolyte chains grafted to nanovector and DNA molecules can have important impacts on the endocytosis.

In this study, the mechanism of single-stranded polynucleotide encapsulation by chitosan and interaction between different types of polynucleotide and chitosan with atomic details were investigated by MD simulations. Herein, three extreme cases for chitosan with 10 units were considered for simplicity: 0% deacetylated structure (designated as –NHCOCH_3_ chitosan), fully deacetylated structure in neutral solution (–NH_2_ chitosan), and fully deacetylated structure in low pH solution (–NH_3_^+^ chitosan). Besides, four types of single-stranded polynucleotide models were used (3 or 10 units of polycytidylic acid and polyguanylic acid) to understand the effect of base and chain length of polynucleotide on the encapsulation by chitosan. These polynucleotides were represented as C3, G3, C10 and G10 in the following sections of the paper. The choice of single-stranded polynucleotides in this work mainly based on two facts: (1) the importance of single-stranded oligonucleotides in gene editing and gene therapy; (2) single-stranded polynucleotide is one of the simplest DNA structure, and it was selected to understand the fundament mechanism of interaction between chitosan and DNA via molecular dynamics simulation. The structure of chitosan and single-stranded polynucleotides with atomic details can be found in Figs S1 and S2 in Supplementary Information.

## Simulation methods

Firstly, the structure and topology of polyglucose with 10 units were generated by LEaP module from the AMBER^33^ suite of programs. Secondly, the initial structures of chitosans with 10 units were generated by replacing the glucose unit –OH group bonded with C2 into –NH_2_, –NH_3_^+^ and –NHCOCH_3_ groups in our home made script (see Fig. S1 in Supplementary Information). The geometry of unit of chitosan with different groups was optimized with HF/6–31G+ by Gaussian03 software^34^. Then the force field parameters of –OH group in glucose topology were replaced by the parameters (including the atom charges, bonded and non-bonded parameters) of –NH_2_, –NH_3_^+^ and –NHCOCH_3_ groups in this home made script. These force field parameters of chitosan unit with different groups were derived from the general AMBER force field (GAFF) and the standard AMBER 2003 force field, respectively, by using AmberTools^33^. The parameters for bonds, angles and dihedral angles between different units of chitosan were obtained from the reference^35^. The partial charges were derived by the calculation of chitosan unit with different functional groups using restrained electrostatic potential (RESP) method at the HF/6-31G+ level in Gaussian 03.

The initial structure of single-stranded polynucleotides was obtained from one chain of standard B-form double-stranded DNA constructed by the nucleic acid database in the Hyperchem software (Version 7.0, Hypercube, Inc). In B-form dsDNA, there is ~10.5 bp in one turn, and extends 34 Å per 10 bp of sequence. The inclination of base pair to axis is around −1.2° and mean propeller twist is +16°. The parameters of polynucleotides were derived from the Amber99sb-ildn force field^36^. All simulations were performed by GROMACS 5.0.7 package^37^. In each system, certain number of chitosans and polynucleotides chains with different ratio (shown in Table 1) were solvated in a box of TIP3P^38^ water molecules by packmol^39^ software. Counter-ions (Na^+^ or Cl^−^) were added into the solution to neutralize the systems. Periodic boundary conditions and a time step of 2 fs were applied. The cut-off of the van der Waals (vdW) interaction was set to be 1.2 nm. The LINCS algorithm^40^ was used to constrain all the bond lengths. The particle mesh Ewald (PME) method^41^ was used to calculate the electrostatic interactions with a cut-off of 1.2 nm. The Lorentz-Berthelot rule^42^ was employed to calculate the Lennard-Jones (LJ) potential of cross interaction between chitosan and polynucleotide. Temperature coupling using velocity rescaling^43^ with a stochastic term was used to keep the temperature at 300 K. The Berendsen method^44^ was used to control the pressure at 1 bar with an isothermal compressibility of 4.5 × 10^−5^ bar^−1^. In each system, a 50000 step energy minimization was firstly performed by steepest descent method^45^. Subsequently, a 200 ps pre-equilibration under an NVT ensemble was performed. Finally, a 100 ns MD simulation in the NPT ensemble was carried out. Visual molecular dynamics (VMD) graphics software was used for molecular visualization^46^.

**Table 1:** All performed simulations in this study.

MD simulations					
Name of system	Number of chitosan	Number of polynucleotides	Type of counter-ions	Number of counter-ions	Simulation time (ns)
NH_3_^+^_C3	6	20	Cl^−^	20	100
NH_2__C3	6	20	Na^+^	40	100
NHCOCH_3__C3	6	20	Na^+^	40	100
NH_3_^+^_G3	6	20	Cl^−^	20	100
NH_2__G3	6	20	Na^+^	40	100
NHCOCH_3__G3	6	20	Na^+^	40	100
NH_3_^+^_C10	6	6	Cl^−^	6	100
NH_2__C10	6	6	Na^+^	54	100
NHCOCH_3__C10	6	6	Na^+^	54	100
NH_3_^+^_G10	6	6	Cl^−^	6	100
NH_2__G10	6	6	Na^+^	54	100
NHCOCH_3__G10	6	6	Na^+^	54	100

## Results and Discussion

### Effect of chitosan’s functional groups on DNA encapsulation

**Figure 2:**
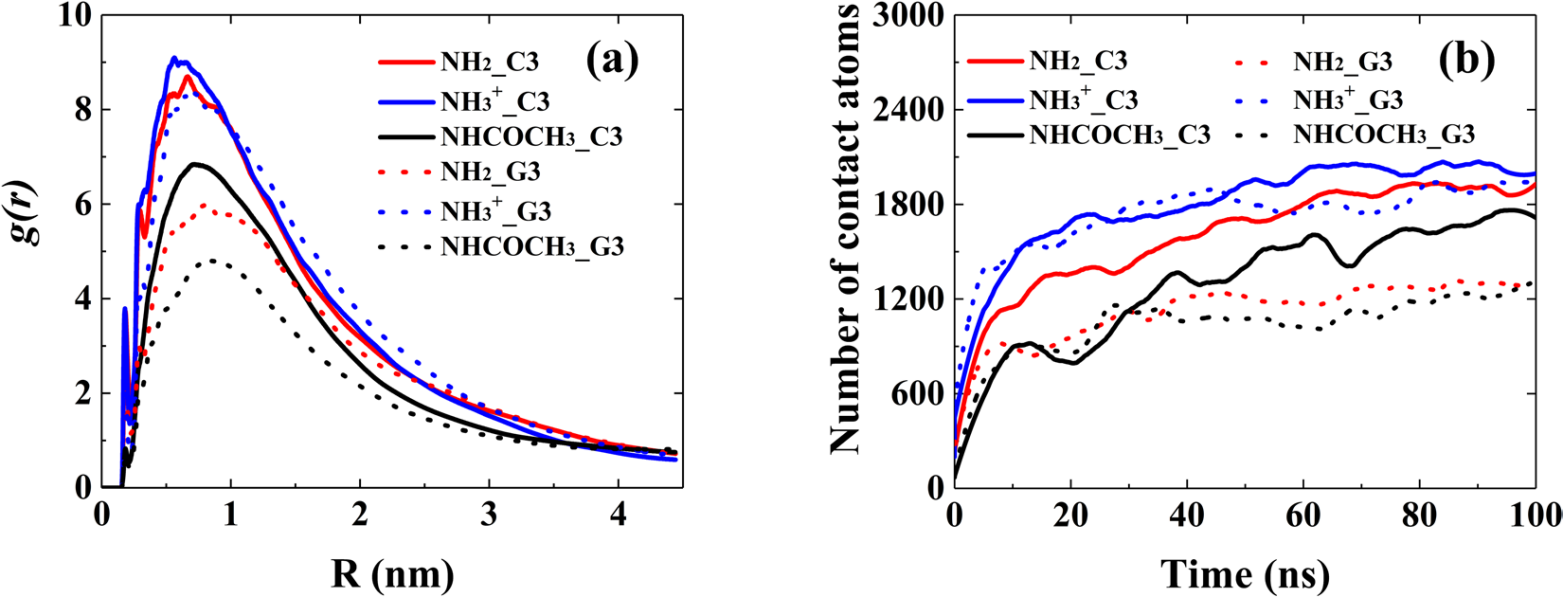
(**a**) The RDFs of C3 and G3 around different functional groups in chitosan (**b**) Number of contact atoms between chitosan and C3/G3 as a function of simulation time, with the ratio chitosan:C3/G3 = 3:10.

**Figure 3:**
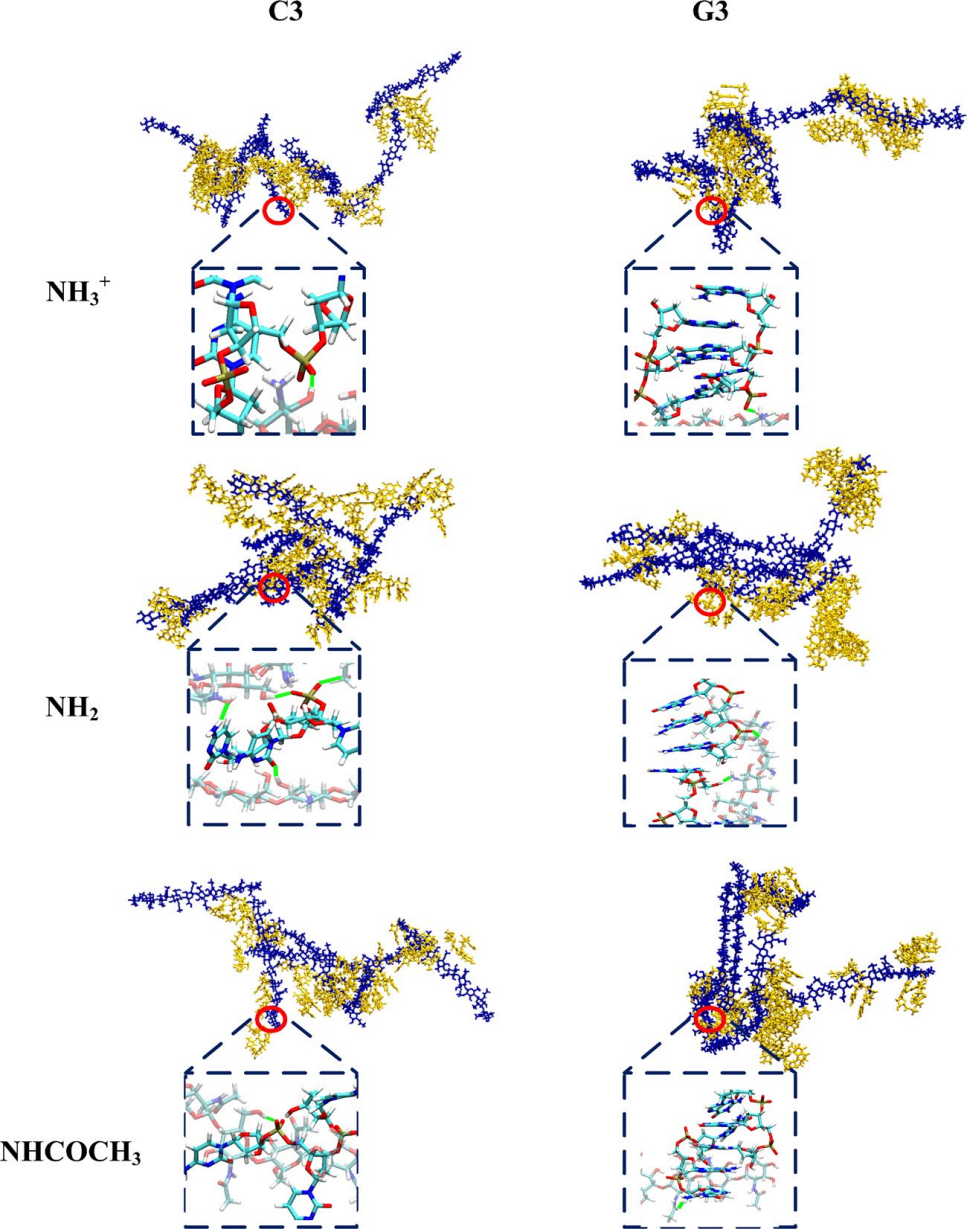
The snapshots of C3 and G3 encapsulated by different types of chitosan at the end of 100 ns MD simulation (blue licorice model: chitosan, yellow licorice model: C3 or G3). Water molecules were omitted for clarity. Enlarged atomic pictures of interaction between chitosan and single-stranded polynucleotides were displayed in blue dashed box (transparent licorice model: chitosan, non-transparent licorice model: C3 or G3, blue: nitrogen, white: hydrogen, red: oxygen, cyan: carbon and orange: phosphorus). The hydrogen bonds formed between chitosan and C3/G3 were marked by solid green line.

Figure 2(a) shown the radial distribution functions (RDFs) of C3 and G3 around different functional groups of chitosan (with the ratio of chitosan:C3/G3 = 3:10, see Table 1). The RDFs was calculated between the center of mass of chitosan’s functional groups (e.g., –NH2, –NH_3_^+^ and –NHCOCH_3_, as shown in Fig. 1) and any atoms of single-stranded polynucleotides. It could be used to quantitatively estimate the distribution of polynucleotides around chitosan and evaluated the effect of chitosan’s functional groups on polynucleotides distribution. The peak height of *g*(*r*) of –NH_3_^+^ chitosan/C3 system (solid blue line) is around 9.1, and it is highest comparing with other systems. It indicates that C3 tends to tightly absorbed on the –NH_3_^+^ chitosan, due to the strong electrostatic attraction between negatively charged C3 and protonated amines of –NH_3_^+^ chitosan. Besides, one could find that the peak height of *g*(*r*) is about 8.9 in –NH_2_ chitosan/C3 system (solid red line). It is relatively high and very close to that of –NH_3_^+^ chitosan system, with slightly narrow distribution of C3 within 2 nm around –NH_2_ chitosan. The peak height of *g*(*r*) is around 6.9 in –NHCOCH_3_ chitosan/C3 system (solid black line), and it is much lower than that of other two types of chitosan complexed with C3. The encapsulation of polynucleotides was also characterized by monitoring the number of intimate contact atoms between chitosan and C3/G3. Herein, an intimate contact between chitosan and C3/G3 was considered if any C3/G3 atoms are within the distance of 6.0 Å of the center of mass of chitosan’s functional groups (e.g., –NH_2_, –NH_3_^+^ and –NHCOCH_3_, as shown in Fig. 1). The time evolution of the number of contact atoms between chitosan and C3/G3 was shown in Fig. 2(b), where one could find more C3 atoms around –NH_3_^+^ chitosan systems. The contact curve have quite similar trend with the results of RDFs curve. Moreover, these differences can also be found in the typical snapshots of these systems at the end of 100 ns MD simulation displayed in Fig. 3. The trajectory and snapshot showed that C3 dispersed into –NH_2_ chitosan, leading to a sandwich-like conformation, while –NHCOCH_3_ chitosan prefer to aggregate together and free C3 can be found distribute around the big condensates of –NHCOCH_3_ chitosan. Figures S3–S8 displayed the polynucleotide encapsulation process in chitosan-C3/G3 systems. These conformations of different chitosan are in accordance with the results of RDFs curve and contact curve, and C3 can be well encapsulated in both –NH_3_^+^ and –NH_2_ chitosan systems.

**Figure 4:**
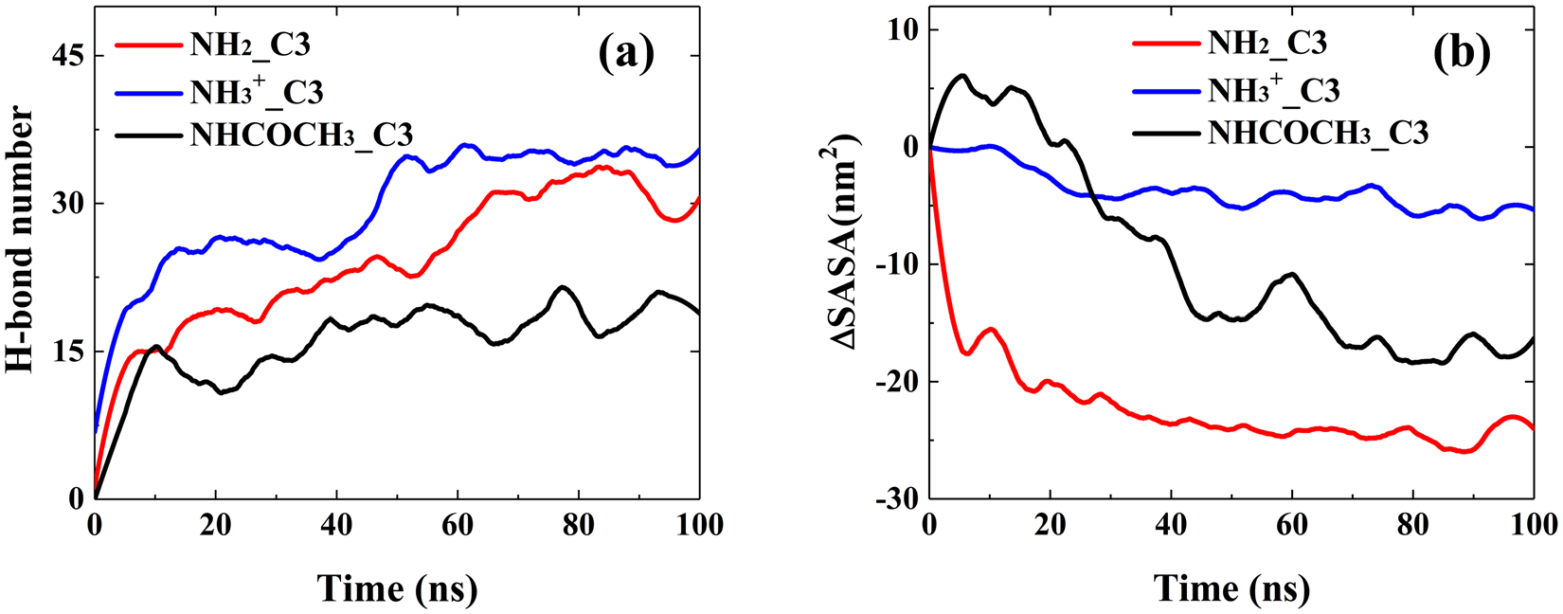
(**a**) The number of inter-molecular H-bonds between different functional groups and C3. (**b**) The SASA change of different types of chitosan in chitosan/C3 systems versus the simulation time.

**Figure 5:**
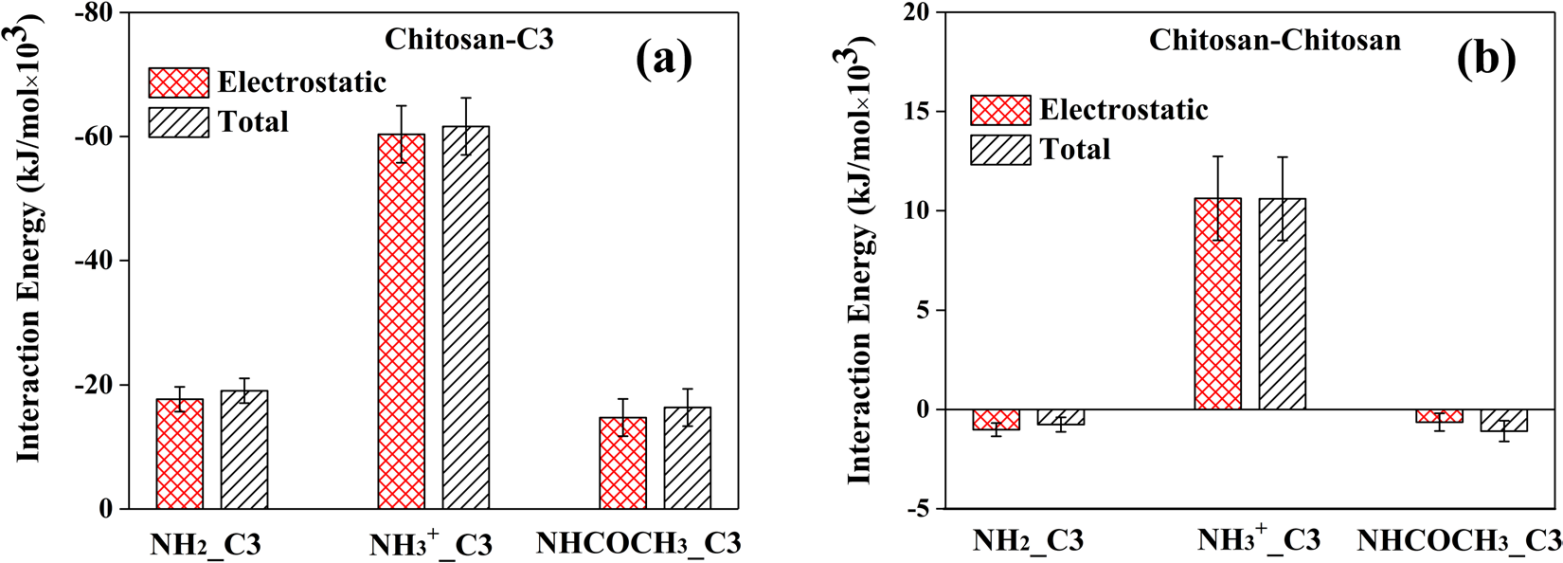
The average electrostatic and total interaction energies of (**a**) Chitosan–C3 and (**b**) Chitosan–chitosan in the systems with the ratio chitosan: C3 = 3:10. Data were taken from the last 50 ns trajectories of the total 100 ns in the MD simulations. The error bars represent the standard deviations of the data.

To better understand the effect of functional group on the encapsulation of polynucleotides, the mechanism of the C3 adsorption onto different types of chitosan was studied. As claimed in several experimental and theoretical studies, hydrogen bond is the main driving force in chitosan structural changes^47, 48^. Herein, the number of inter-molecular H-bond in C3 system was calculated to address the effect of functional groups. Figure 4(a) showed that the number of H-bond formed between C3 and different functional groups increased as a function of simulation time and slightly fluctuated at the end of the simulation. One could find that the system of –NH_3_^+^ chitosan/C3 has the largest H-bond numbers among three systems, which indicates that the strength of H-bond between C3 and –NH_3_^+^ chitosan is strongest. It could be attribute to the strong electrostatic interaction between positive charged –NH_3_^+^ group in chitosan and negatively charged –PO_4_^2−^ group in C3, as shown in Fig. 5(a). With tight contact between –NH_3_^+^ group in chitosan and –PO_4_^2−^ in C3, the chance of forming H-bond is high. Figure 5(a) also showed that the electrostatic interaction dominates the interaction between C3 and different types of chitosan. In the AMBER force field, there is no special term that is specific to hydrogen bonds. The hydrogen bond energy still arises from the dipole–dipole interaction of the donor and acceptor groups, and it is added to the electrostatic potential. Therefore, H-bond has great contribution to the electrostatic interaction and total interaction energy. It has large effect on the interaction strength between C3 and chitosan, and thus affects the distribution of C3 around chitosan.

In Fig. 4(b), the solvent accessible surface area (SASA) change of different types of chitosan versus the simulation time was displayed. Comparing with other two systems, –NH_3_^+^ chitosan has smallest change of SASA. Due to the inter-molecular and intra-molecular repulsive electrostatic interaction between –NH_3_^+^ groups, –NH_3_^+^ chitosans have more stretched chain conformations and less aggregate structure. Both –NH_2_ and –NHCOCH_3_ chitosan has lower SASA value at the end of simulation, and the notable change of SASA in these two systems suggests that they could form more aggregated and condensed structure. To confirm this argument and provide a reference for dynamics of aggregation of –NH_2_ and –NHCOCH_3_ chitosan, the SASA change of different types of chitosan without polynucleotides during 100 ns MD simulation were plotted in Fig. S9. It was found that without polynucleotide the SASA of both of –NH_2_ and –NHCOCH_3_ chitosan remarkably decreased. These results consist with the fact that deacetylation could increase the solubility of chitosan, and the solubility of chitosan at low pH (most of the –NH_2_ groups are protonated) is much higher^49^.

The difference of the RDFs curve for –NH_2_ and –NHCOCH_3_ chitosan systems in Fig. 2(a) can also be explained by comparing the H-bond numbers and ∆SASA curve. Note that C3 in –NH_2_ chitosan/C3 system can form much higher number of inter-molecular H-bond with –NH_2_ chitosan than that with –NHCOCH_3_ chitosan/C3 system, and this could be attributed to the fact that –NH_2_ group have more H-donors than –NHCOCH_3_ group. Therefore, –NHCOCH_3_ chitosan have weaker interaction energy with C3 comparing with –NH_2_ chitosan (see Fig. 5). In addition, the strong tendency of –NH_2_ chitosan’s self-assembly will tightly encapsulate C3, leads to a sandwitch-like morphology of –NH_2_ chitosan/C3 system and increase the distribution of C3 around –NH_2_ chitosan. On the basis of strong interaction (including H-bond) between C3 and –NH_2_ chitosan, although –NH_2_ chitosan could form stronger aggregated structure than –NH_3_^+^ chitosan, the distribution of C3 around –NH_2_ chitosan is slightly lower than that around –NH_3_^+^ chitosan.

### Effect of base type on DNA encapsulation

As reported in several studies^50, 51^, the nature of polynucleotides has great influence on their interaction with gene carriers. For instance, compared with DNA, the siRNA was able to establish stronger interactions with carriers due to its more flexible structure^50^. Herein, for the purpose of understanding the effect of base types on DNA encapsulation, the process of G3 encapsulation into different types of chitosan with the ratio chitosan: G3 = 3:10 were also investigated (see Table 1).

As shown in Fig. 2(a), the peak height of RDF in –NH_3_^+^ chitosan/G3 system (blue dot line) is around 8.2, and it is a little bit lower than that in –NH_3_^+^ chitosan/C3 system. However, in the case of –NH_2_ (red dot line) and –NHCOCH_3_ (black dot line) chitosan/G3 systems, the peak height of the RDFs curve of G3 is much lower, which indicates that the distribution of G3 around these two types of chitosan is much lower than that of C3. These results can also be found by comparing the difference of number of contact atoms in these systems, as shown in Fig. 2(b). For further comparison, the typical snapshots of different types of chitosan complexed with C3 and G3 at the end of 100 ns MD simulation were also displayed in Fig. 3. It suggested that in –NH_2_ and –NHCOCH_3_ chitosan/G3 systems, the aggregation of G3 is more obvious than that of C3 in solution. It is not surprising that guanine has larger conjugated ring than that of cytosine, and the aggregation of G3 in solution is stronger. To better address the effect of different bases, 20 C3 and G3 molecules in solution were separately simulated for 80 ns in absence of chitosan, and the typical snapshots during the aggregation process for two polynucleotides were displayed in Fig. S10 (see Supplementary Information). Figure S10 suggested that G3 have stronger tendency of aggregating comparing with C3. This is the reason that in both –NH_2_ and –NHCOCH_3_ systems, G3 has less distribution around chitosan. However, due to the strong electrostatic interaction (including high strength of H-bonds) between –NH_3_^+^ groups in chitosan and –PO_4_^2−^ groups in nucleotides, the aggregation of both C3 and G3 could be highly suppressed, and both of them could well disperse around the positively charged –NH_3_^+^ chitosan. In other words, the electrostatic interaction is the driving force for the interaction between –NH_3_^+^ chitosan and polynucleotides.

**Figure 6:**
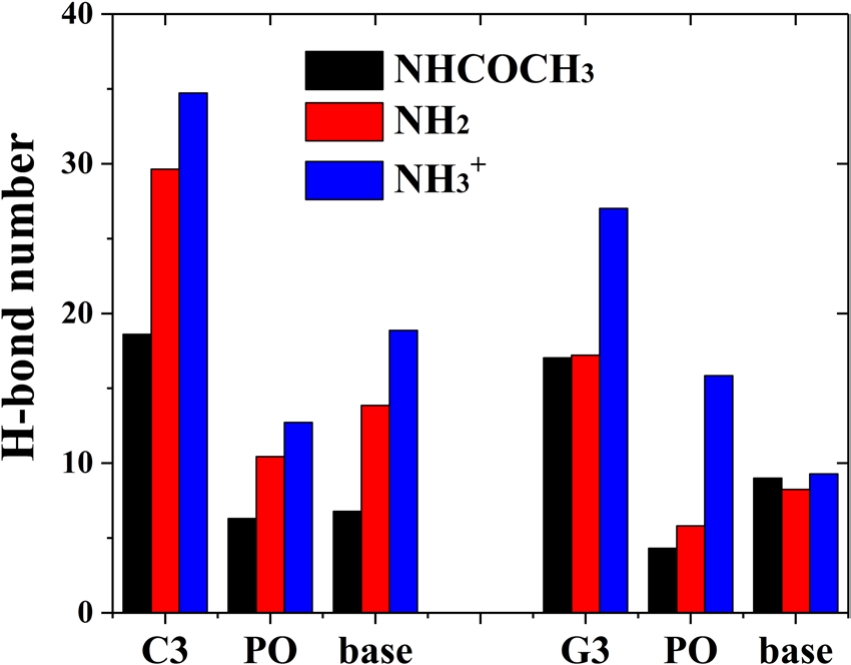
The average number of inter-molecular H-bond between different functional groups and different part of C3 and G3 during the MD simulations (C3/G3: total number of H-bond formed between chitosan and polynucleotides; PO: phosphate; base: guanine or cytosine).

Since the polynucleotides are composed of nitrogenous base, five-carbon sugar and phosphate group, the H-bond number between the functional groups in three chitosan and different part of polynucleotides were calculated to extend the analysis with more chemical detail, as shown in Fig. 6. Due to the aggregation of G3, the number of H-bond decreased for all three systems comparing with the systems containing C3. Moreover, one could find that the number of H-bond between G3 and –NH_2_/–NHCOCH_3_ chitosan is quite small, which may also cause by strong aggregation of G3. The higher number of H-bond between G3 and –NH_3_^+^ chitosan primarily arise from the H-bond formed between –NH_3_^+^ group and phosphate group. This fact confirms the significance of electrostatic interaction in polynucleotide encapsulation by chitosan.

In short summary, compared with C3, due to stronger stacking ability of base, G3 have stronger tendency of aggregating in the solution, and this property may decrease the polynucleotide encapsulation capacity by chitosan and following delivery efficiency. For –NH_3_^+^ chitosan, due to the strong electrostatic interaction, especially the H-bond between –NH_3_^+^ group in chitosan and phosphate group in polynucleotide, the aggregation effect could be partially eliminated. Thus the encapsulation capacity of polynucleotide by chitosan is mainly balanced by two factors: the strength of polynucleotide binding to chitosan and the tendency of self-aggregation of polynucleotide.

### Effect of chain length on polynucleotide encapsulation

In this section, the effect of chain length on polynucleotide encapsulation was investigated. Six systems with polynucleotide chain length of 10 (C10 or G10) and different types of chitosan were constructed and simulated (see Table 1). The molecular ratio of chitosan: C10/G10 = 1:1.

**Figure 7:**
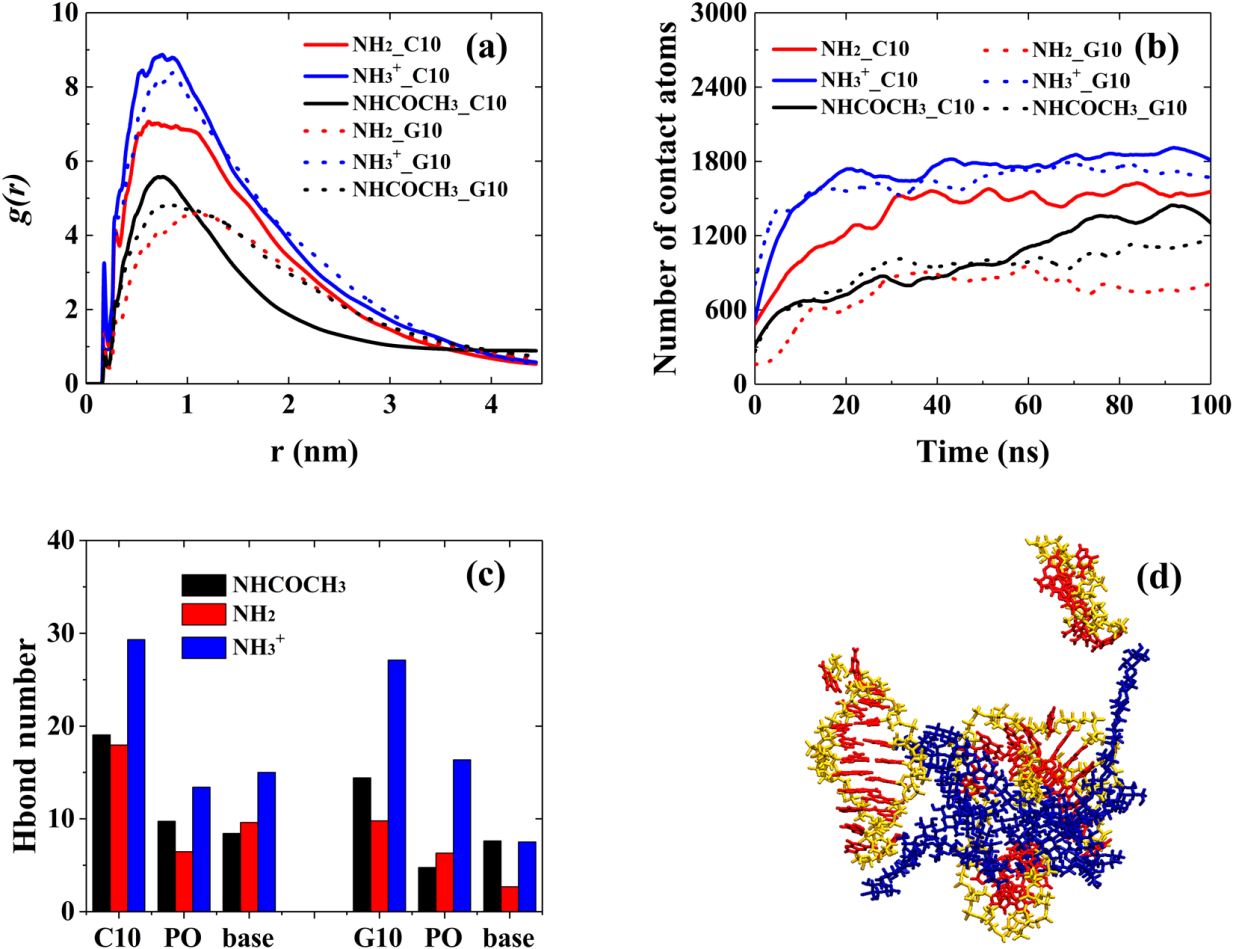
(**a**) The RDFs of C10/G10 around different types of chitosan. (**b**) Number of contact atoms between chitosan and C10/G10 as a function of simulation time. (**c**) Inter-molecular H-bond numbers between functional groups in chitosan and different parts of nucleotide (C10/G10: total number of H-bond formed between chitosan and C10/G10; PO: phosphate; base: guanine or cystosine). (**d**) The snapshots of –NH2 chitosan/G10 system at the end of 100 ns MD simulation (blue: chitosan, yellow: G10, red: base). All bases in G10 were displayed in red to better understand the role of base-base interaction in aggregation of G10. Water molecules were omitted for clarity.

Figure 7(a) showed the RDFs of C10/G10 around different types of chitosan. For –NH_3_^+^ chitosan system, increasing the chain length of polynucleotide has very little effect on the distribution of polynucleotide around chitosan: C3 (see Fig. 2(a)) has slightly higher density and closer distance around –NH_3_^+^ chitosan than C10. However, for –NH_2_ and –NHCOCH_3_ chitosan, chain length has large effect on the polynucleotide encapsulation. For example, both C3 and G3 have larger and closer distribution around –NH_2_ chitosan (see Fig. 2(a)), comparing with C10 and G10. The number of contact atoms in these systems, as shown in Fig. 2(b) and Fig. 7(b), also confirmed the results derived from RDFs. If one carefully check the equilibrated structure of –NH_2_ chitosan-polynucleotide complexes, it was interesting that part of the long chain polynucleotide could stack to each other or even keep certain distance from chitosan, as shown in Fig. 7(d). Figure S11–S16 showed the polynucleotide encapsulation process in chitosan-C10/G10 systems. In the cases of –NH_2_ chitosan systems, the capability of C10 and G10 dispersed along the chitosan chain was highly decreased. This phenomenon probably origins from (1) relative flexible conformation of C10 and G10 and (2) more base-base stacking sites of C10 and G10. Similar to G3 aggregation, the stacking of polynucleotide with chain length of 10 would decrease the interaction strength between polynucleotide and chitosan, and thus decrease the distribution of polynucleotide around chitosan. This could also be reflected by H-bond number between C10/G10 and functional groups in chitosan, as shown in Fig. 7(c). The number of H-bond between –NH_2_ groups and C3/G3 (see Fig. 6) greatly decreased with chain length increase, which indicates the decreased interaction strength. Meanwhile, the number of H-bonds between –NH_3_^+^ chitosan and polynucleotide with different chain length has little difference, which consist with the RDFs curves. This phenomenon could attribute to the fact that strong interaction between positively charged –NH_3_^+^ and negatively charged phosphate groups could minimized the effect of base stacking of C10 and G10.

We need add a cautious remark that in this study we only considered three extreme cases for chitosan: 0% deacetylated structure, fully deacetylated structure in neutral solution, and fully deacetylated structure in low pH solution. Although the model is relatively simple, it is the early stage of investigating chitosan-DNA interaction at the molecular level by molecular simulation. Further consideration of structure of double-stranded DNA, different degree of deacetylation of chitosan, different pH environment and hydrophilic/hydrophobic modification of chitosan etc. will be taken in our future studies to help understand the gene/drug encapsulation and delivery by chitosan-based delivery systems.

## Conclusion

In this study, molecular dynamics simulations were performed to investigate the effects on single-stranded polynucleotide encapsulation by chitosan and the underlying interaction mechanism between polynucleotide and different types of chitosan with atomic details. It was found that the functional groups of chitosan, the types of base and length of polynucleotides regulate the interaction behavior between chitosan and polynucleotides. The encapsulation capacity of polynucleotide by chitosan is mainly balanced by two factors: the strength of polynucleotide binding to chitosan and the tendency of self-aggregation of polynucleotide in the solution. For –NH_3_^+^ chitosan, due to the strong electrostatic interaction, especially the H-bond between –NH_3_^+^ group in chitosan and phosphate group in polynucleotide, the aggregation effect could be partially eliminated. Therefore, both C3/G3 and C10/G10 in –NH_3_^+^ chitosan have better dispersal capacity than that in –NH_2_ and –NHCOCH_3_ chitosan. The good dispersal capacity of polynucleotides may improve the encapsulation of polynucleotides by chitosan, and hence increase the delivery and transfection efficiency of gene carrier. Our results revealed the atomic interaction mechanism between polynucleotide and various types of chitosan, and may provide novel ideas for the design and application of chitosan-based drug and gene delivery systems.

## Electronic supplementary material

Supplementary Information
