# Supplementary material for: Molecular dynamics study on the mechanism of polynucleotide encapsulation by chitosan
Source: Sci Rep. 2017 Jul 11;7:5050. doi: 10.1038/s41598-017-05197-0 (PMC5506017; doi:10.1038/s41598-017-05197-0)
Supplement: Supplementary file 1 — Supplementary Information [file 41598_2017_5197_MOESM1_ESM.docx]

**Supplementary Information**

**Molecular dynamics study on the mechanism of polynucleotide encapsulation by chitosan**

Jia-Wei Shen^1,#,^*, Jiachen Li^2,#^, Zhennan Zhao^1^, Li Zhang^2,^*, Guoteng Peng^2^, Lijun Liang^3,^*

^1^School of Medicine, Hangzhou Normal University, Hangzhou 310016, People’s Republic of China

^2^Department of Chemistry, Key Laboratory of Advanced Textile Materials and Manufacturing Technology of Education Ministry, Zhejiang Sci-Tech University, Hangzhou, 310018, People’s Republic of China

^3^College of Life Information Science and Instrument Engineering, Hangzhou Dianzi University, Hangzhou, 310018, People’s Republic of China

^#^ These authors contributed equally to this work.

* Corresponding authors.

Tel: +86-571-2886-5674; Fax: +86-571-2886-9344 (J.W. Shen)

E-mail addresses: [shen.jiawei@hotmail.com](mailto:shen.jiawei@hotmail.com), [lizhang@zstu.edu.cn](mailto:lizhang@zstu.edu.cn), [michael.lijunl@gmail.com](mailto:michael.lijunl@gmail.com)


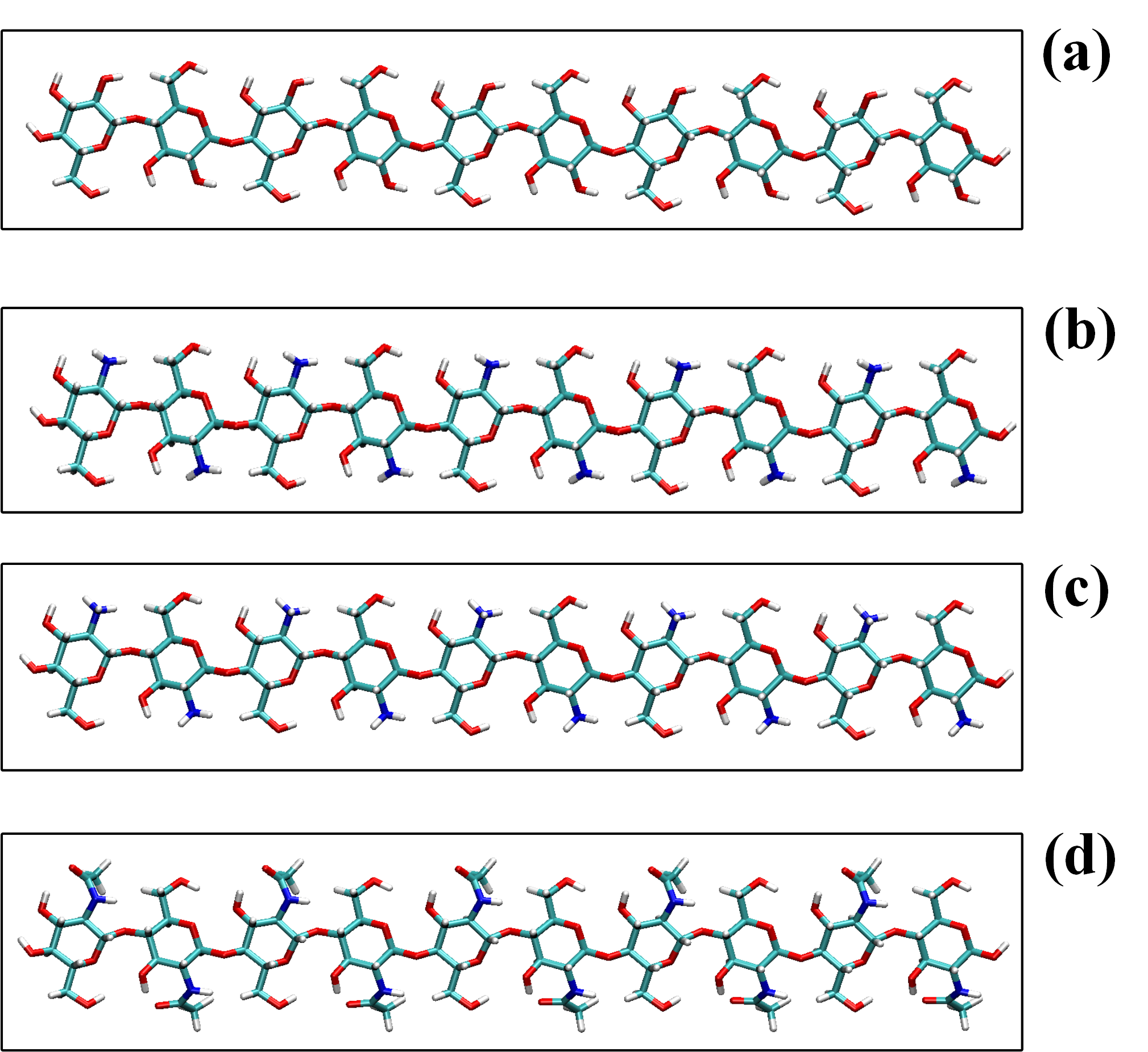


**Fig. S1.** The initial structure of chitosan with 10 units was generated by replacing the glucose unit –OH group bonded with C2 into –NH_2_, –NH_3_^+^ and –NHCOCH_3_ groups. (a) polyglucose, (b) –NH_2_ chitosan, (c) –NH_3_^+^ chitosan and (d) –NHCOCH_3_ chitosan. All atoms were shown in licorice model (blue: nitrogen, white: hydrogen, red: oxygen, cyan: carbon and orange: phosphorus).


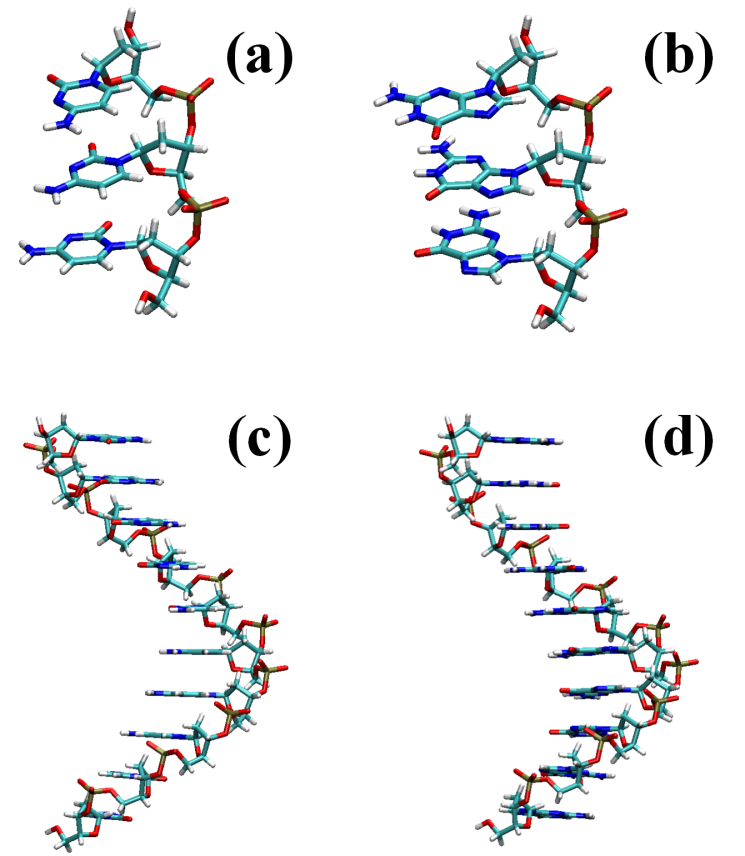


**Fig. S2.** The structures of single-stranded polynucleotides with atomic details (a) C3, (b) G3, (c) C10 and (d) G10. All atoms were shown in licorice model (blue: nitrogen, white: hydrogen, red: oxygen, cyan: carbon and orange: phosphorus).


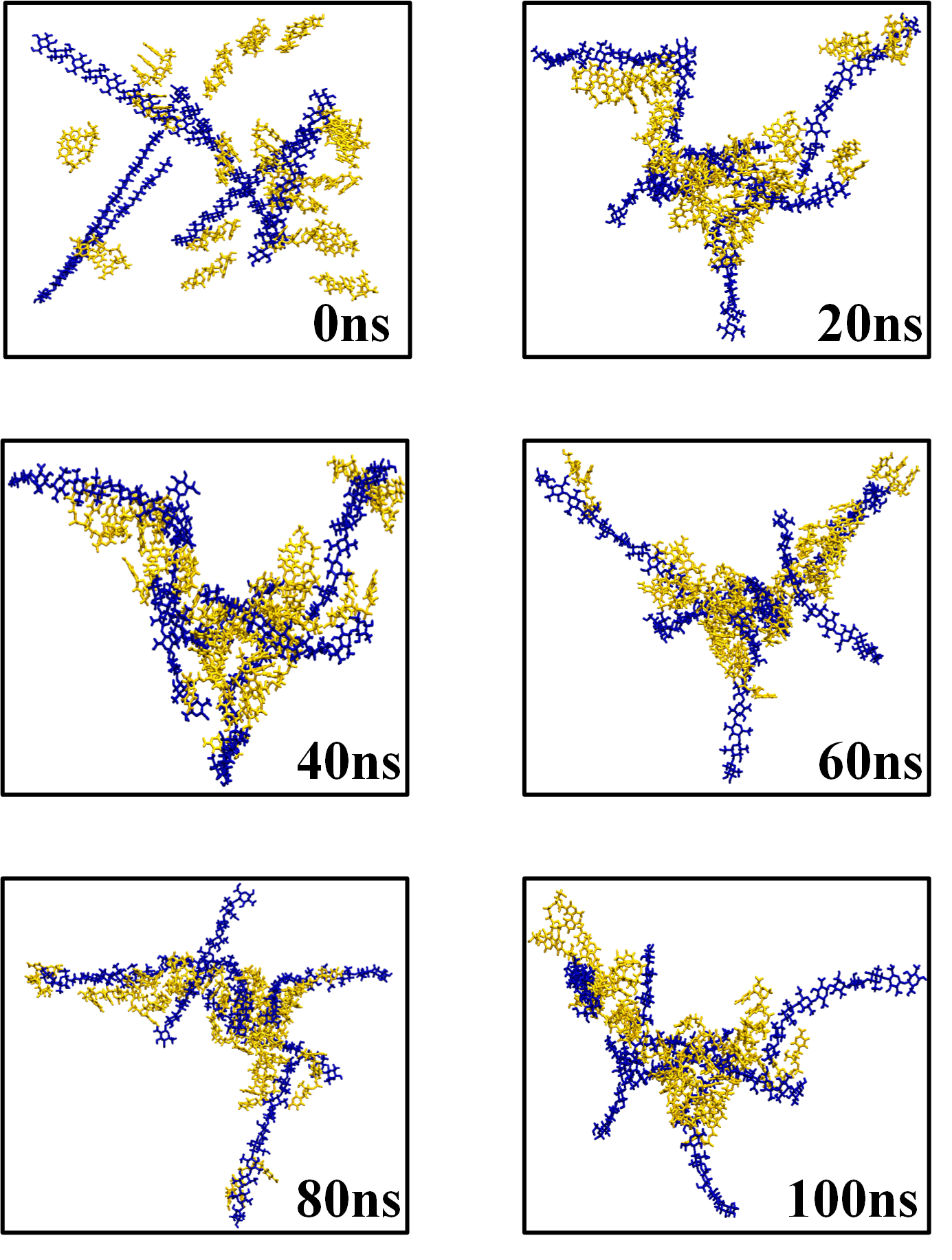


**Fig. S3.** Typical snapshots during the encapsulation process of –NH_3_^+^ chitosan/C3 system (blue licorice model: chitosan, yellow licorice model: polynucleotides). Water molecules were omitted for clarity.


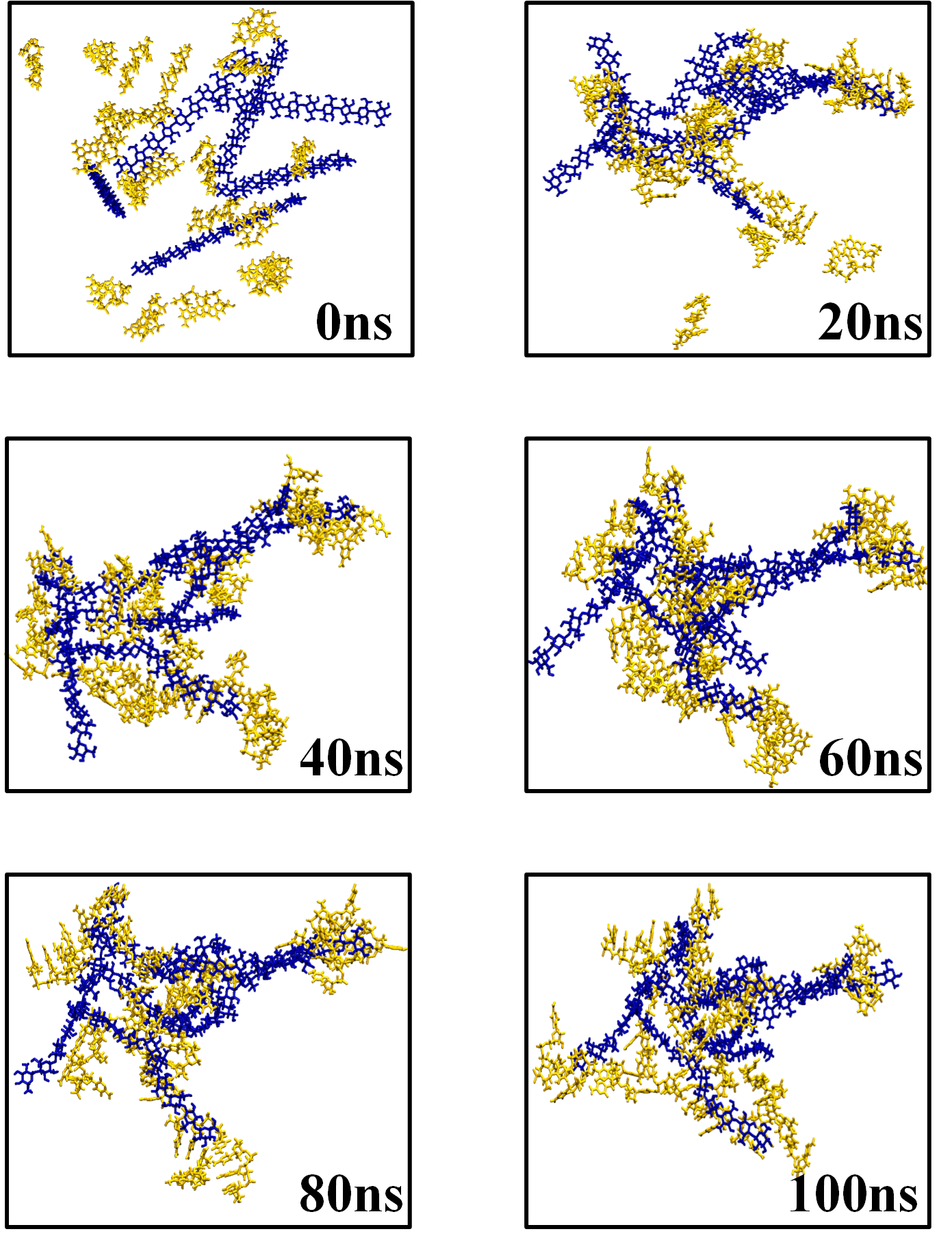


**Fig. S4.** Typical snapshots during the encapsulation process of –NH_2_ chitosan/C3 system (blue licorice model: chitosan, yellow licorice model: polynucleotides). Water molecules were omitted for clarity.


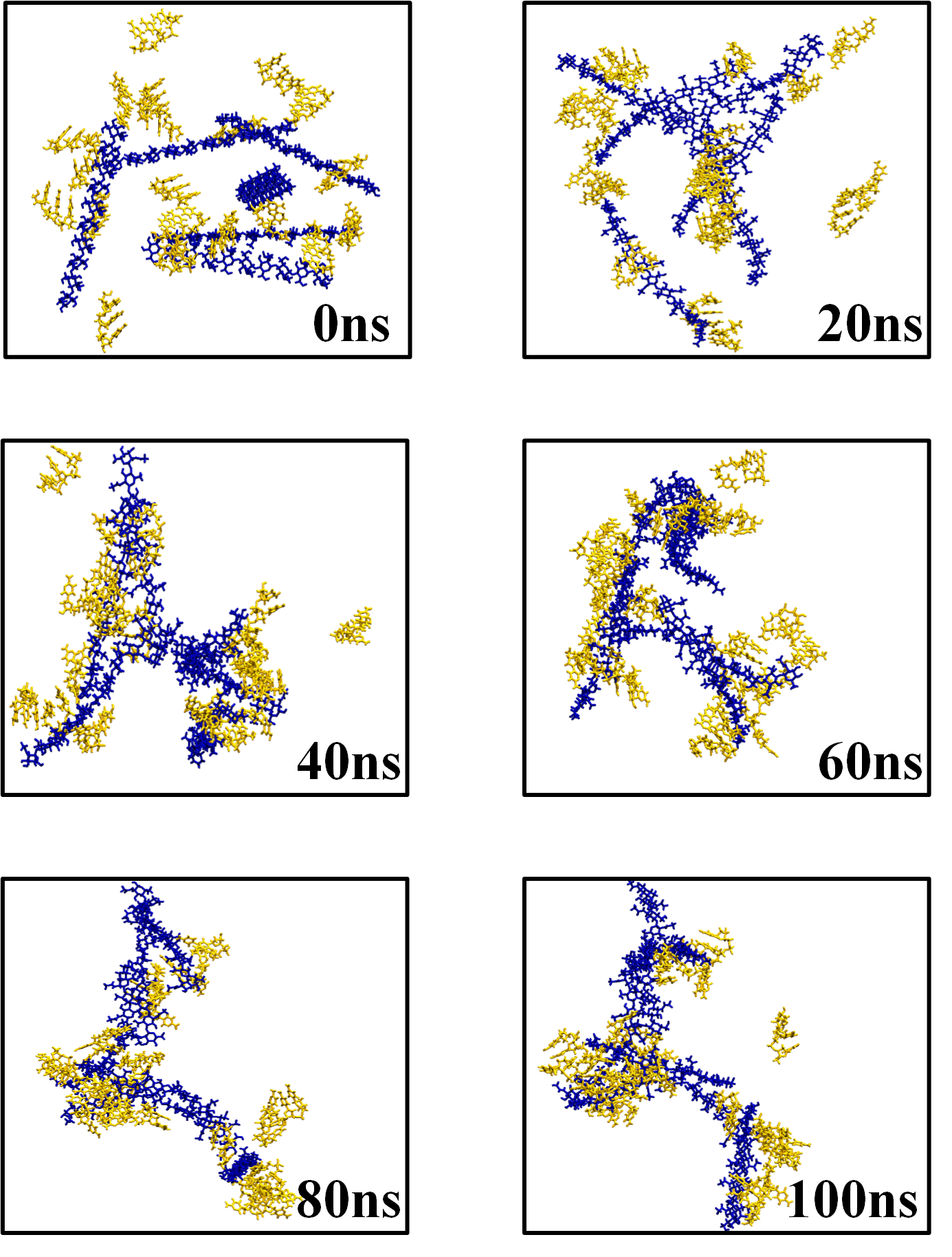


**Fig. S5.** Typical snapshots during the encapsulation process of –NHCOCH_3_ chitosan/C3 system (blue licorice model: chitosan, yellow licorice model: polynucleotides). Water molecules were omitted for clarity.


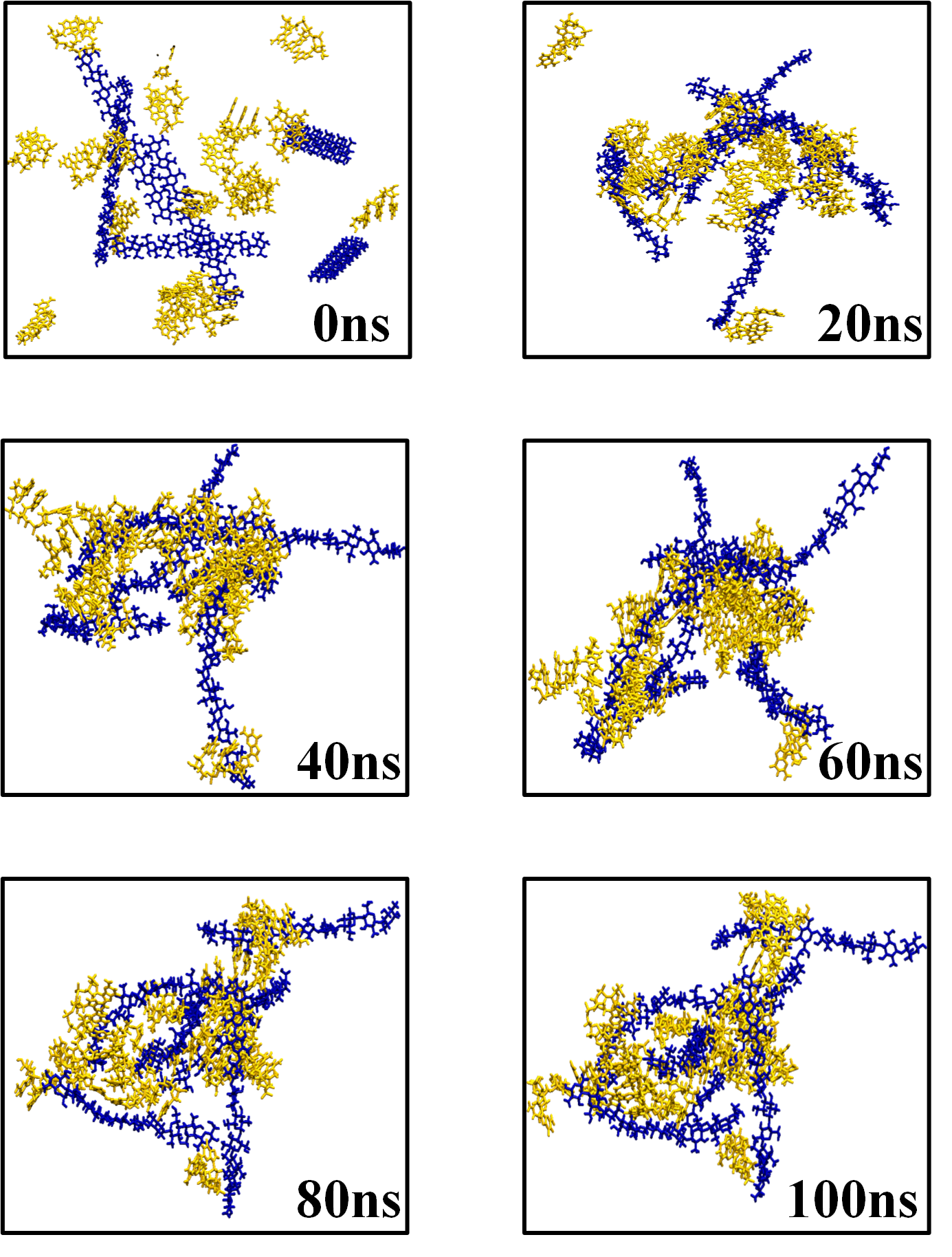


**Fig. S6.** Typical snapshots during the encapsulation process of –NH_3_^+^ chitosan/G3 system (blue licorice model: chitosan, yellow licorice model: polynucleotides). Water molecules were omitted for clarity.


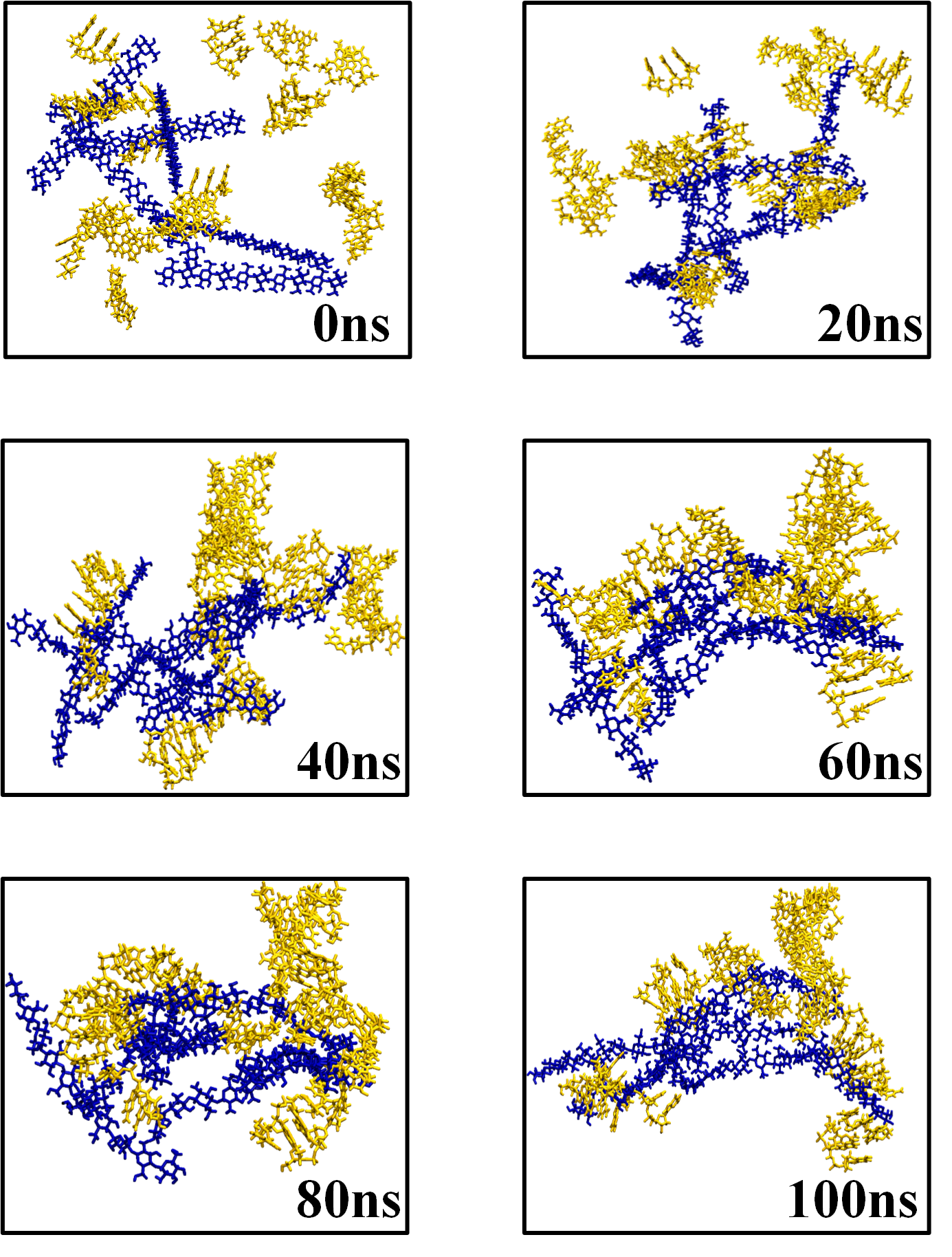


**Fig. S7.** Typical snapshots during the encapsulation process of –NH_2_ chitosan/G3 system (blue licorice model: chitosan, yellow licorice model: polynucleotides). Water molecules were omitted for clarity.


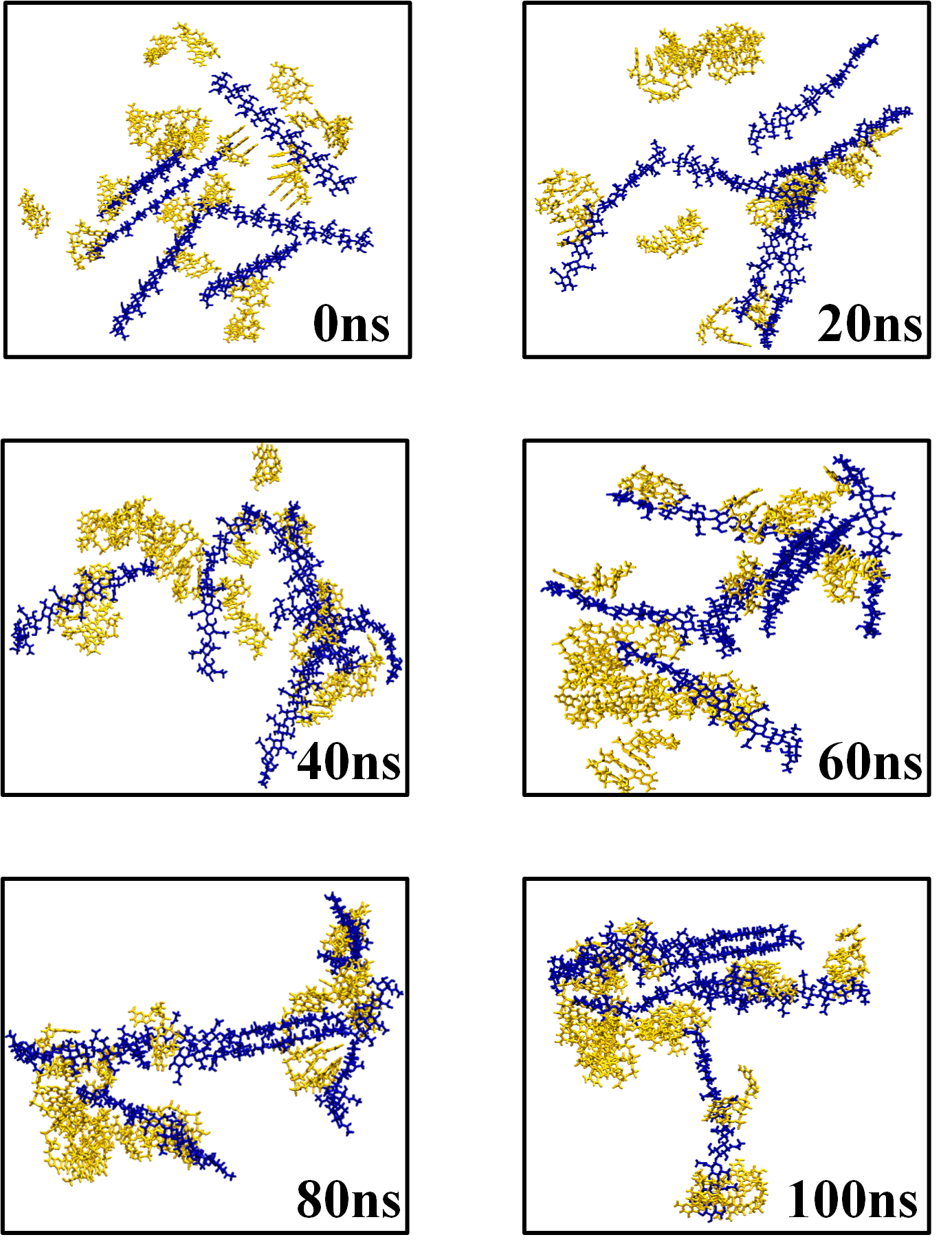


**Fig. S8.** Typical snapshots during the encapsulation process of –NHCOCH_3_ chitosan/G3 system (blue licorice model: chitosan, yellow licorice model: polynucleotides. Water molecules were omitted for clarity.


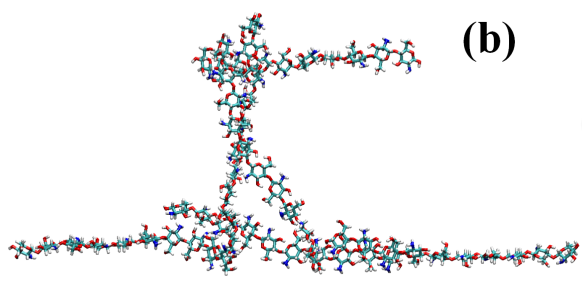

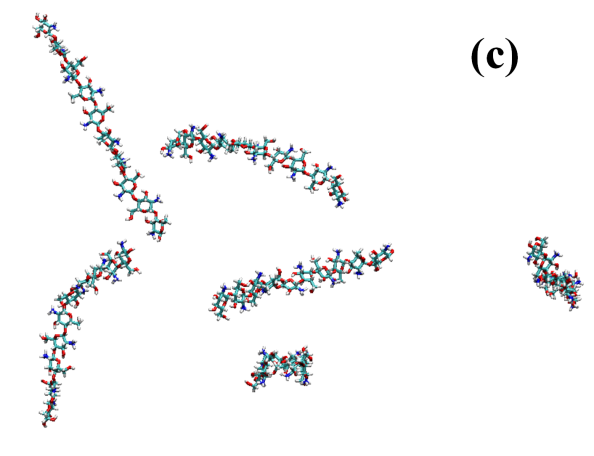


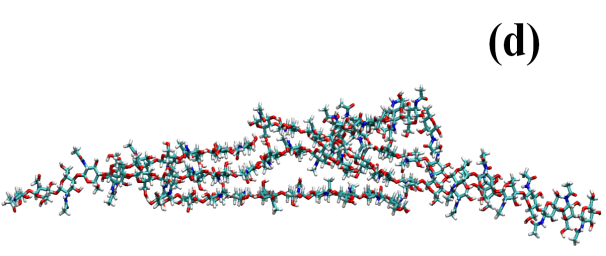


**Fig. S9.** (a) The SASA change of different types of chitosan without polynucleotides in solution during 100ns MD simulation. The structure snapshot of (b) –NH_2_ chitosan, (c) –NH_3_^+^ chitosan and (d) –NHCOCH_3_ chitosan at the end of 100ns MD simulation (blue: nitrogen, white: hydrogen, red: oxygen, cyan: carbon and orange: phosphorus). Water molecules were omitted for clarity.


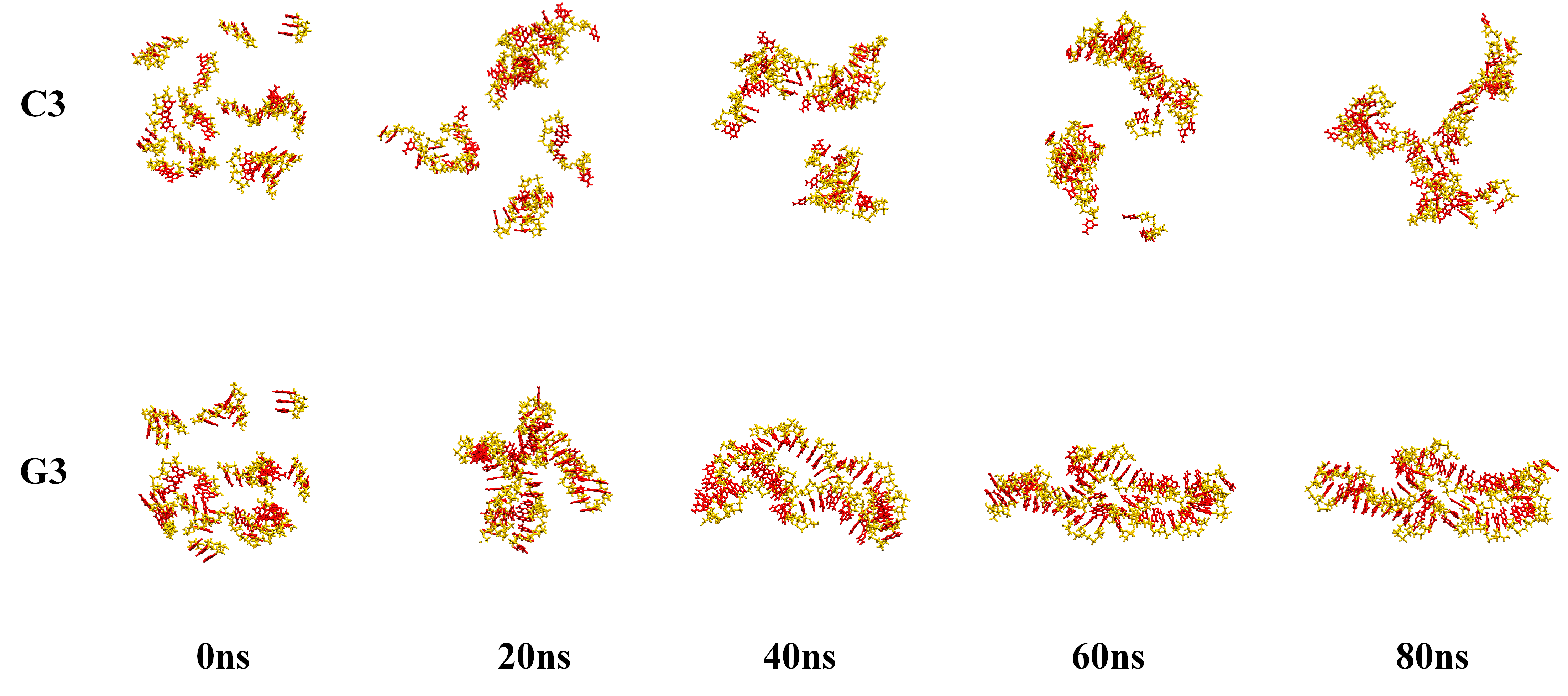


**Fig. S10.** Typical snapshots during the aggregation process of C3 and G3 in the solution without chitosan. The bases were shown in red licorice model to better understand base-base interaction. The rest parts of polynucleotides were shown in orange licorice model.

**
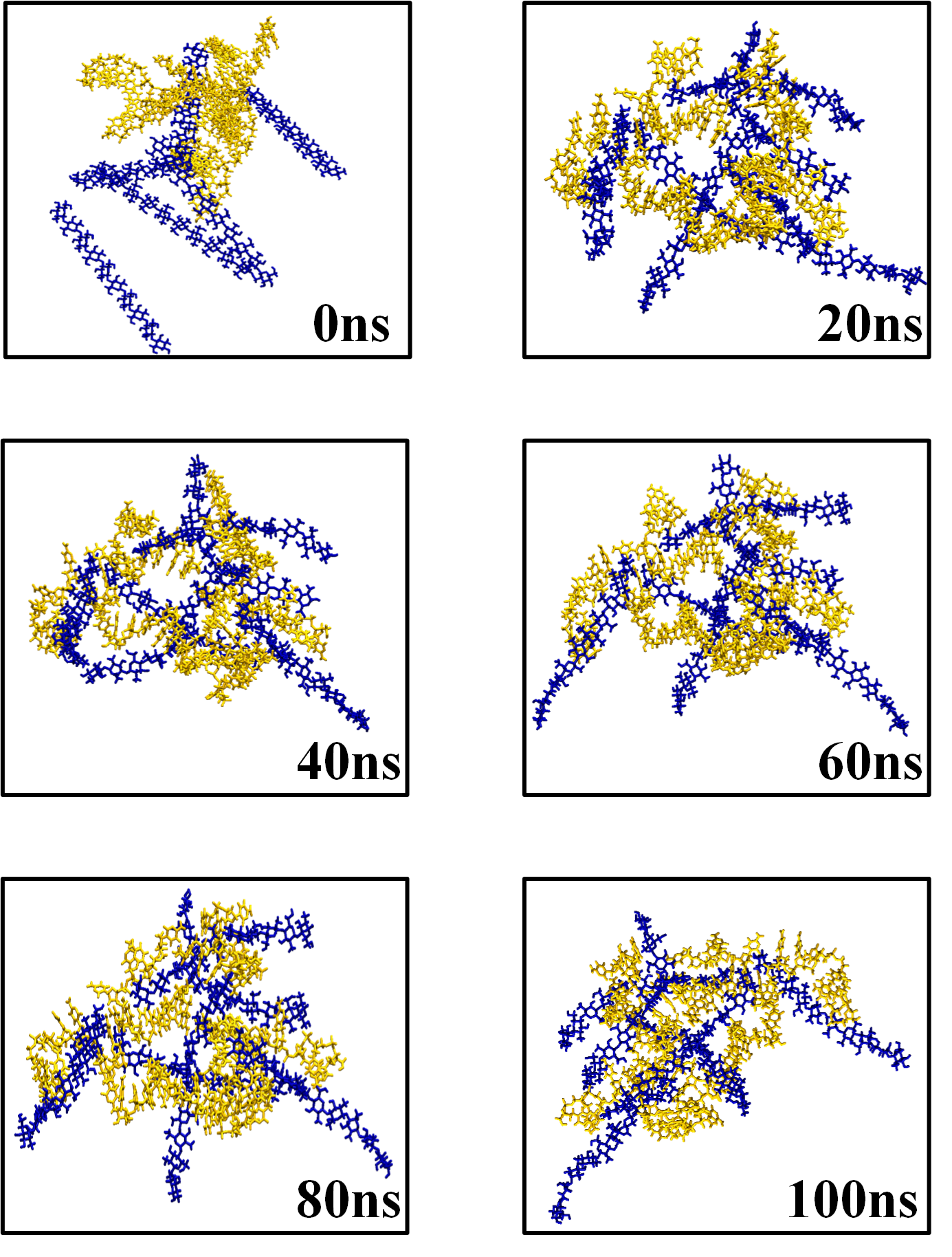
**

**Fig. S11.** Typical snapshots during the encapsulation process of –NH_3_^+^ chitosan/C10 system (blue licorice model: chitosan, yellow licorice model: polynucleotides). Water molecules were omitted for clarity.

**
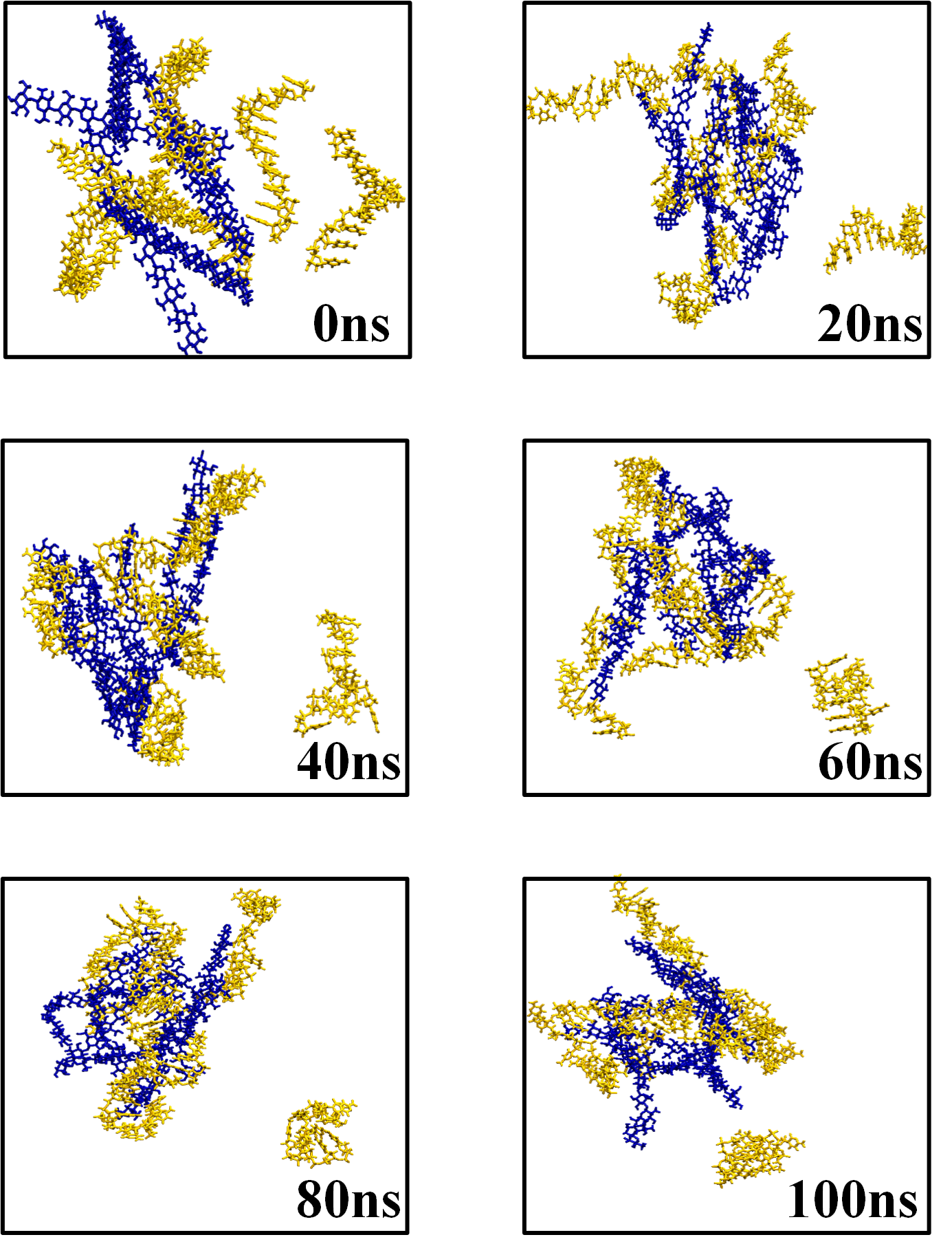
Fig. S12.** Typical snapshots during the encapsulation process of –NH_2_ chitosan/C10 system (blue licorice model: chitosan, yellow licorice model: polynucleotides). Water molecules were omitted for clarity.

**
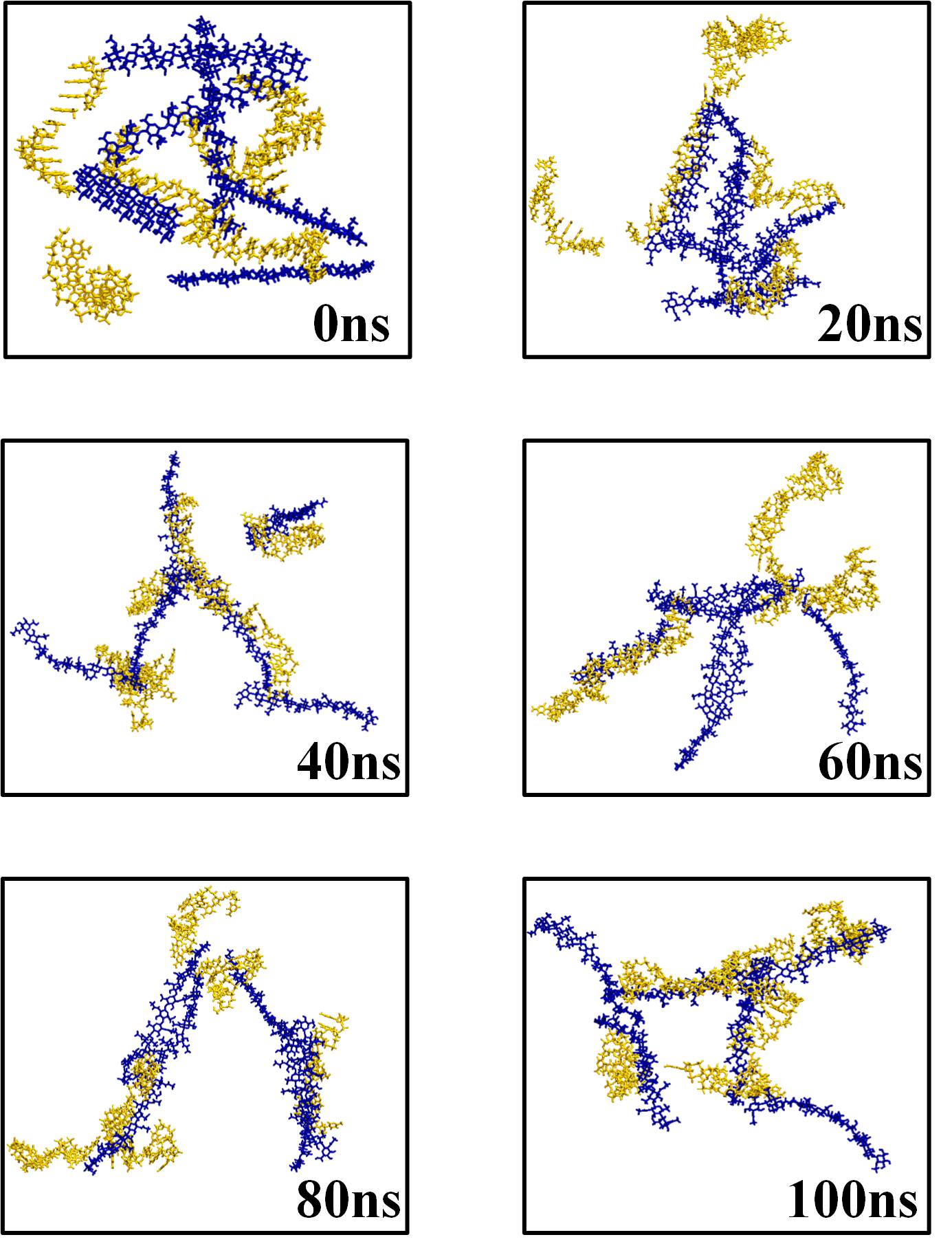
**

**Fig. S13.** Typical snapshots during the encapsulation process of –NHCOCH_3_ chitosan/C10 system (blue licorice model: chitosan, yellow licorice model: polynucleotides). Water molecules were omitted for clarity.

**
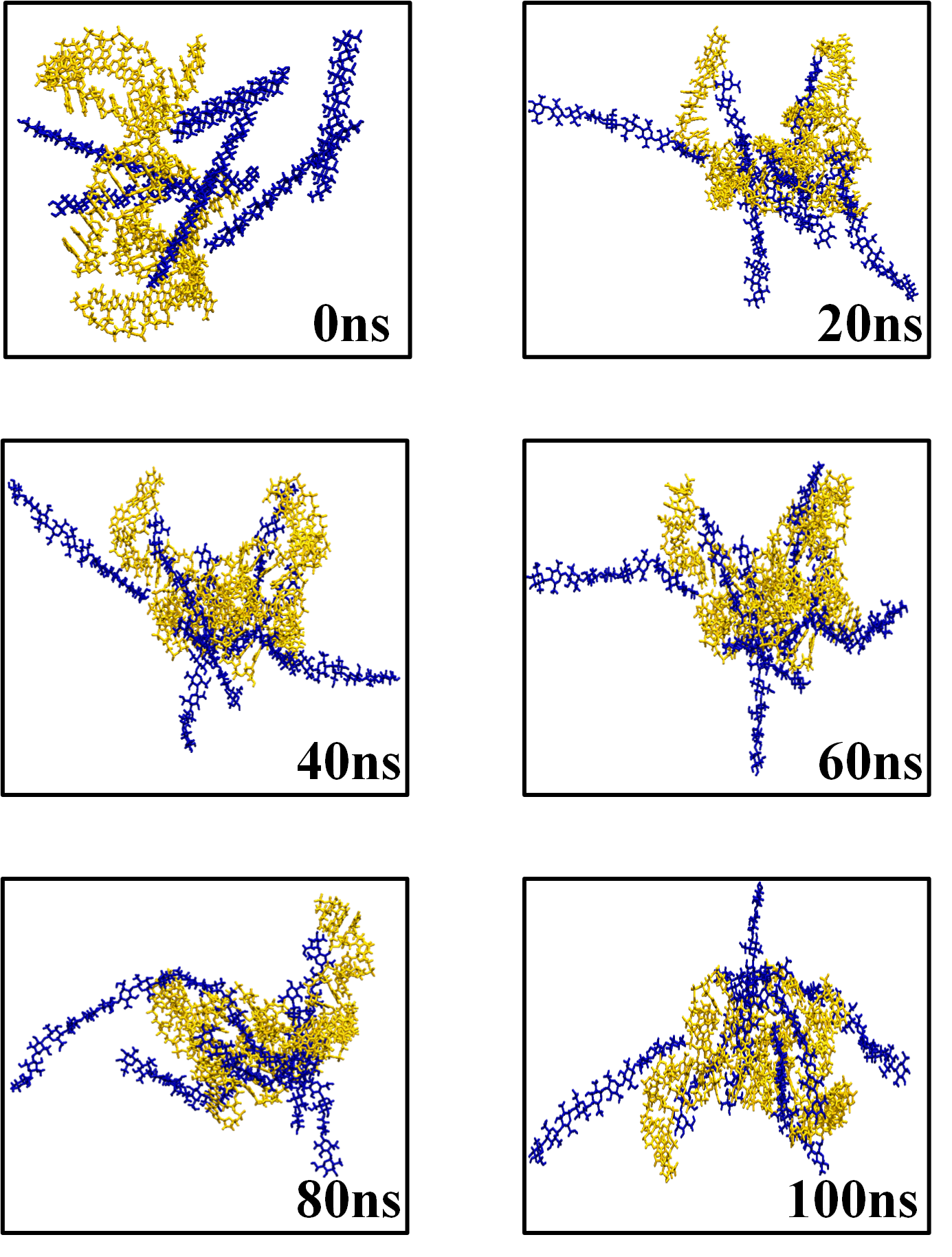
Fig. S14.** Typical snapshots during the dynamics encapsulation of –NH_3_^+^ chitosan/G10 system (blue licorice model: chitosan, yellow licorice model: polynucleotides). Water molecules were omitted for clarity.

**
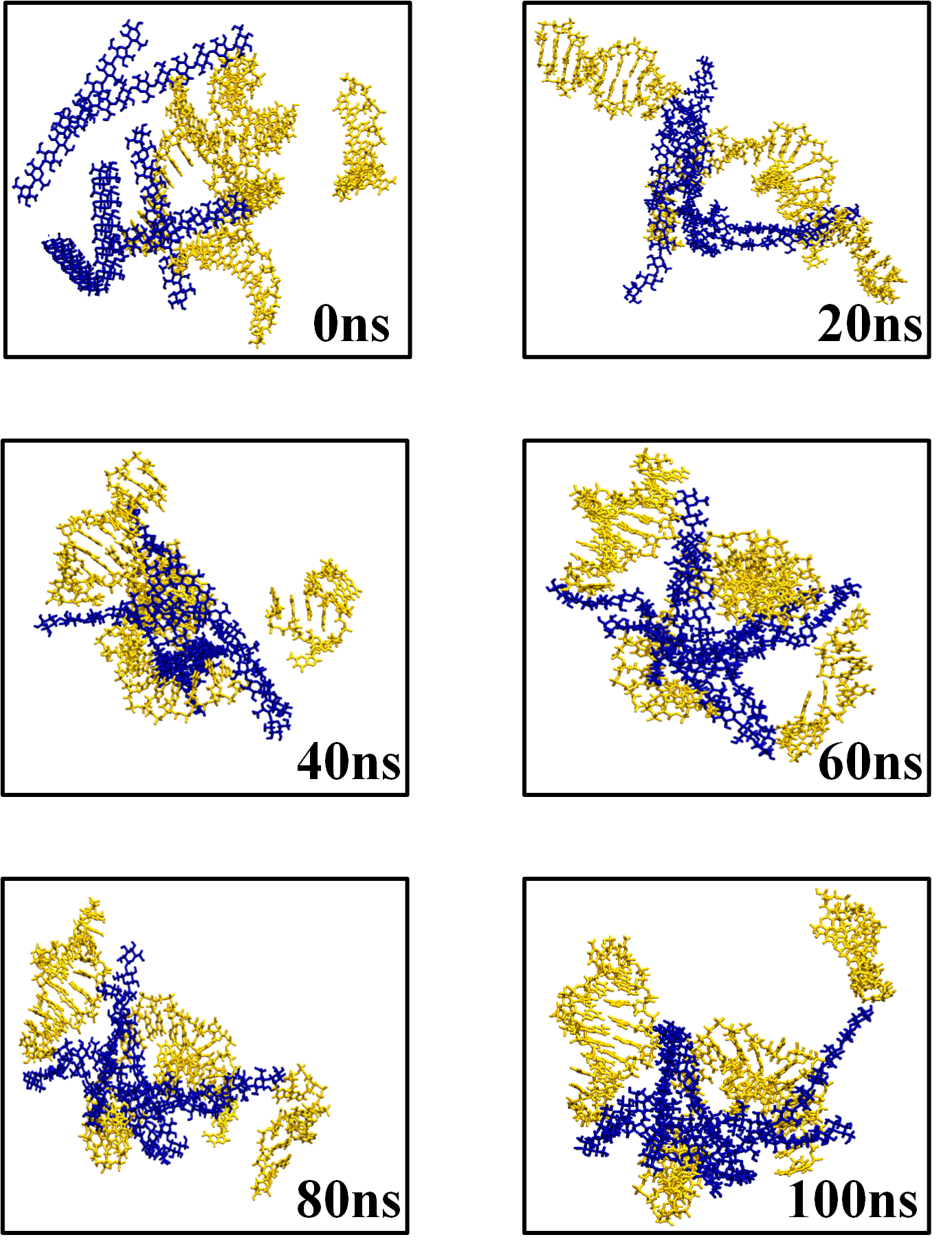
Fig. S15.** Typical snapshots during the encapsulation process of –NH_2_ chitosan/G10 system (blue licorice model: chitosan, yellow licorice model: polynucleotides). Water molecules were omitted for clarity.

**
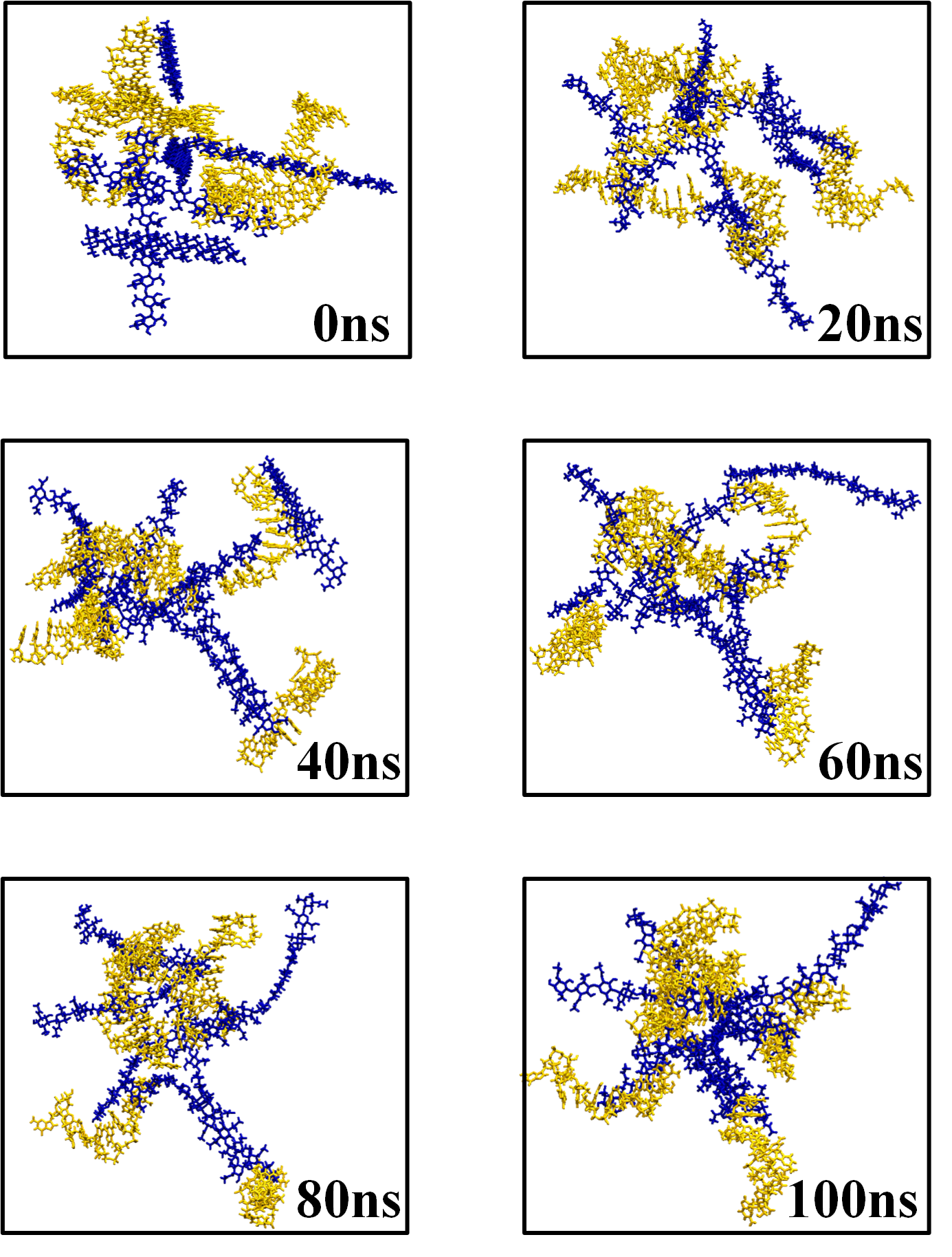
Fig. S16.** Typical snapshots during the encapsulation process of –NHCOCH_3_ chitosan/G10 system (blue licorice model: chitosan, yellow licorice model: polynucleotides). Water molecules were omitted for clarity.
